# The use of oral contraceptives and the risks of developing prehypertension and hypertension in women of reproductive age: findings from a population-based survey in Indonesia

**DOI:** 10.1186/s12889-025-22686-4

**Published:** 2025-04-24

**Authors:** Neily Zakiyah, Aeni Suciati, Chika Aulia Afina, Sofa Dewi Alfian

**Affiliations:** 1 Department of Pharmacology and Clinical Pharmacy, Faculty of Pharmacy, Universitas Padjadjaran, Bandung, Indonesia; 2 Center of Excellence for Pharmaceutical Care Innovation, Universitas Padjadjaran, Bandung, Indonesia

**Keywords:** Oral contraceptive, Prehypertension, Hypertension, Women of reproductive age, IFLS-5

## Abstract

**Background:**

Although the underlying mechanism of the effect of oral contraceptives (OCs) on blood pressure (BP) remains unclear, previous research showed that OCs are associated with an increased risk of elevated BP. This study aims to analyze the association between OCs and the risk of prehypertension and hypertension in women of reproductive age in Indonesia, using longitudinal data from the Indonesian Family Life Survey (IFLS) 5 (2014–2015).

**Methods:**

A cross-sectional study was conducted on 10,279 subjects using data from IFLS-5. The study included all women of reproductive age (15–49 years) with complete data on contraceptive use and BP. Sociodemographic factors, physical activity, dietary habits, depressive symptoms, history of tobacco use, and comorbidities were covariates. Multivariate logistic regression analyses were conducted to estimate odds ratios (OR), along with 95% confidence intervals (95% CI) and p-values, comparing women using OCs to those not using contraceptives and those using other modern contraceptive methods. Sub-analyses were conducted for the duration of OC use.

**Results:**

The results indicated that OC use was associated with a significantly increased risk of both prehypertension and hypertension. The adjusted odds ratios (aOR) for prehypertension and hypertension were 1.42 (95% CI: 1.16–1.73, *p* = 0.001) and 1.72 (95% CI: 1.45–2.05, *p* < 0.001), respectively, compared to non-users. When compared to users of other modern contraceptive methods, the aORs for prehypertension and hypertension were 1.74 (95% CI: 1.21–2.51, *p* = 0.003) and 1.80 (95% CI: 1.31–2.48, *p* < 0.001). Sub-analyses showed that the odds of hypertension increased with longer durations of OC use, but no significant association was found for prehypertension.

**Conclusion:**

There is a statistically significant moderate increase in the risk of prehypertension and hypertension among women of reproductive age in Indonesia who use OCs, with the risk of hypertension increasing with longer durations of OC use. This finding highlights the need for healthcare providers to carefully assess risks when prescribing contraceptives, particularly for women with cardiovascular risk factors.

**Supplementary Information:**

The online version contains supplementary material available at 10.1186/s12889-025-22686-4.

## Introduction

Prehypertension and hypertension contribute significantly to the global disease burden worldwide, and it is one of the major risk factors for cardiovascular diseases (CVDs), particularly coronary heart disease and stroke [1–4]. Despite several improvements in the prevention and management of CVDs, the incidence and number of CVDs-related mortality in women remain high [5], indicating the need to assess specific gender-specific risk factors. In alignment with most major guidelines, including those applicable in Indonesia, hypertension is defined as a systolic blood pressure (SBP) of ≥ 140 mm Hg and/or diastolic blood pressure (DBP) of ≥ 90 mm Hg, while prehypertension refers to an SBP between 120 and 139 mmHg or a DBP between 80 and 89 mmHg [6, 7].

The prevalence of prehypertension and hypertension has increased globally [8–10]. In Indonesia, it is estimated that the prevalence of prehypertension is around 22.5% in the young population (20–30 years) and approximately 32.5% in older adults (> 40 years) [11, 12]. Additionally, the prevalence of hypertension in the Indonesian population 18 years or older was 25.8% in 2013 and increased to 34.11% in 2018, with a higher proportion in women than in men [13]. Hypertension in women results from interactions between genetic and environmental factors, such as a family history of hypertension, overweight or obesity, increased salt and alcohol intake, and other environmental-related aspects, including the use of OCs [14, 15].

OCs remain among the most popular and widely used effective contraceptive methods globally. It is estimated that 151 million women of reproductive age (15–49 years) worldwide use OCs [16]. Previous studies showed that the use of OC is associated with elevated blood pressure (BP), an underlying cause of hypertension [14, 15, 17, 18]. Although the exact mechanism is unclear, the estrogen and progestin content may interfere with the renin-angiotensin-aldosterone system, which regulates BP [19, 20].

Although OCs are considered a possible risk factor for hypertension, there is currently relatively limited information available on the effect of OC use on BP in women of reproductive age in Indonesia. Findings from other populations may not fully capture Indonesia’s unique contraceptive use patterns, genetic predispositions, and environmental factors. Indonesia has one of the highest OC usage rates in Southeast Asia, with combined OCs being the most commonly used hormonal contraceptive. Unlike some countries where long-term methods dominate, Indonesia’s contraceptive trends have shifted, with short-term methods remaining prevalent despite a recent decline [21, 22]. Factors such as maternal age, urban residence, socioeconomic status, education, employment, and number of children significantly shape contraceptive choices. These variations may influence OC use patterns and their potential impact on BP [23]. Genetic factors also play a role in hypertension, with certain gene mutations linked to elevated BP being more common in Asian populations, including Indonesia. These genetic predispositions may affect the relationship between OC use and hypertension differently compared to other regions [24, 25]. Additionally, environmental factors, such as dietary habits, physical activity levels, and socioeconomic status, can significantly influence hypertension risk. In Indonesia, rapid urbanization and lifestyle changes have led to an increased prevalence of hypertension [26]. These environmental changes, combined with unique cultural and socioeconomic factors, may interact with OC use differently than in other populations, potentially affecting the association between OC use and (pre)hypertension [25].

Given that OCs are among the most commonly used contraceptive methods in Indonesia [27–29], even a small increased risk of side effects may have a large clinical and burden impact. Therefore, this study aimed to explore the association between OC use and the risk of prehypertension and hypertension among Indonesian women of reproductive age, utilizing data from the Indonesian Family Life Survey (IFLS) 5 (2014–2015). By focusing on this specific population, the study seeks to address existing gaps in the literature and provide regionally relevant insights into the relationship between OC use and BP.

## Methods

This study adheres to the Strengthening the Reporting of Observational Studies in Epidemiology (STROBE) guidelines for reporting observational studies, as outlined in Supplementary File 1.

### Data sources and study design

We obtained the data sources for this study from population-based national data from the latest waves of IFLS 5 (2014–2015). IFLS is a large-scale longitudinal demographic household survey conducted in Indonesia since 1993. It includes publicly available data at the individual, household, community, and facility levels. The multistage stratified sampling design was used in the survey and represents 83% of the Indonesian population, as samples were obtained from 13 provinces in Indonesia, including four provinces on Sumatra Island, five provinces on Java, and four provinces in East Indonesia. The latest waves were even broader and included more than 20 provinces to consider respondents’ mobility to other provinces. The IFLS questionnaire underwent preliminary pilot testing to confirm its reliability and validity before the full-scale survey was conducted. The research was approved by the RAND Human Subjects Protection Committee’s ethical review board (s0064-06-01-CR01). Prior to data collection, all respondents provided written informed consent [30].

We conducted a cross-sectional study using the latest waves of IFLS 5. The inclusion criteria were women of reproductive age (15–49 years), with complete data on the use of OCs, BP values, and sociodemographic characteristics. We excluded participants with incomplete data on the use of contraception and BP.

### Variable classification

#### Prehypertension and hypertension

The participants’ BP data were collected either through direct measurements using the Omron digital self-inflating sphygmomanometers HEM-7023 m in three consecutive readings or based on a diagnosis from a medical professional, such as a doctor, paramedic, nurse, or midwife [30]. The device provides accurate systolic and diastolic BP readings, with a margin of error of ± 3 mm Hg for BP and ± 5% for pulse rate [31]. Participants were informed about the study protocol, which included three daily BP measurements. The BP measurement database for the IFLS-5 study followed the American Heart Association standards [32]. Before the first measurement, respondents were seated in a reclined chair, with legs uncrossed, feet flat on the floor, and forearms resting at heart level [30]. The first reading was taken at the start of the interview, with the next two measurements recorded throughout the interview. The average was used for the analysis [33]. This information was utilized to determine the frequency of prehypertension and hypertension, which are categorized into three groups: “normal” (systolic BP/ SBP < 130 mmHg and diastolic BP/DBP < 85 mmHg), “prehypertension” (SBP 130–139 or DBP 85–89 mmHg), and “hypertension” (SBP ≥ 140 mmHg or DBP ≥ 90 mmHg [6].

#### The use of oral contraceptive

Data on the use of OCs were obtained using a questionnaire submitted to the respondents, “Are you or your husband currently using a contraceptive method to delay or prevent pregnancy?” with a Yes or No response. Respondents were asked about their current method of contraception with the question: ‘What contraceptive method are you currently using?’ The responses were used to classify contraceptive use into three categories: ‘OC users,’ ‘other users’ (respondents using another modern contraceptive method besides OCs), and ‘non-users’ (respondents not using any contraceptive method). Modern contraceptives include the use of intrauterine devices (IUD), contraceptive injections, contraceptive tubes, or implants, intravag, condom and female condom, tubal ligation/ female sterilization, and vasectomy/ male sterilization. In addition, information on the duration of OC use was gathered through a questionnaire that asked respondents, “When did you first start using this method?” and “When did you (last) use this method?“. The duration was then categorized into three groups: 0–12 months, 12–24 months, and more than 24 months.

#### Sociodemographic characteristics and comorbidities as potential confounding factors

Sociodemographic characteristics, including age, employment status, level of education, residential location, history of tobacco use, physical activity, obesity, dietary habits, depressive symptoms, and comorbidities such as diabetes mellitus, CVDs, and dyslipidemia were collected.

The respondent’s reported age was used to determine the age variable during the survey. Additionally, residential areas were classified as urban or rural. Education levels were based on responses to the question, “What is the highest level of education you have attended?” and categorized into five groups: None, Elementary School/Equivalent, Junior High School/Equivalent, Senior High School/Equivalent, University and Others (for respondents who answered, “Do not know” or “Special School”). Employment status was established by the “Yes” or “No” response to the question, “Did you engage in work activities, attempt to earn income, or assist in earning income during the past week?“. Tobacco use history was assessed through the question, “Have you ever used chewing tobacco, smoked tobacco using a pipe, rolled your own cigarettes, or smoked cigars?” with “Yes” or “No” options.

The obesity variable was derived from the measured weight data (kg) using Camry scale (model EB1003), and height (meters) with Seca plastic height board (model 213) [30]. Body Mass Index (BMI) was calculated from these measurements, and respondents were categorized as “obese” or “non-obese” based on whether their BMI was greater than 26 or less than or equal to 26, respectively, in accordance with the cut-off point for the Indonesian population [34]. Physical activity was assessed using the “International Physical Activity Questionnaire-Short Form (IPAQ-SF)”. Responses were processed according to the IPAQ scoring protocol, and classified into three categories: light, moderate, and heavy [35]. Comorbidity conditions, including diabetes mellitus, CVDs, and dyslipidemia were identified by asking participants whether they had been diagnosed with any of the conditions: diabetes mellitus, high cholesterol, heart attack, coronary heart disease, angina, or other heart-related conditions.

Depressive symptoms were measured using a set of 10 questions that asked respondents, “How have you felt over the past week?“. Respondents indicated how frequently each statement applied to them using a three-point Likert scale (0 = rarely or none of the time, 1 = some or a little of the time, 2 = moderately or much of the time, and 3 = most or almost all of the time). The total score was calculated by summing up all responses, with positive-mood items being reverse-coded. Respondents with a total score of 10 or higher were classified as having depressive symptoms [36].

Dietary habits were assessed through questions such as, “What type of food you usually eat?” and " How many days in a week did you eat […] in the last week?“. The responses were converted into a Food Consumption Score (FCS), following the methodology developed by the World Food Programme [37].

### Statistical analysis

Descriptive analyses were conducted to determine the distribution and characteristics of respondents’ data on each variable, reported as total numbers (N) and percentages (%). Sociodemographic characteristics and comorbidities were compared between three BP level groups (normal, prehypertension, and hypertension) with a chi-square test. Variables with a significance level of *p* < 0.25 in univariate analyses were included in the multivariate analysis. Binary logistic regression was performed to obtain the crude and adjusted odds ratio (cOR and aOR, respectively), 95% CI (confidence interval), and p-value. R-squared was also estimated to indicate the extent to which the combination of independent variables simultaneously explains the variation in the dependent variable. Additionally, a sub-analysis was conducted to evaluate the association between prehypertension and hypertension with the duration of OC use. This analysis was performed within a single binary logistic regression model, where OC duration was treated as a categorical variable (short-term, medium-term, long-term). Comparisons were made between OC users, users of other modern contraceptive methods, and non-users. The analysis was carried out using STATA version 17 statistical software, and a p-value < 0.05 is considered statistically significant.

## Results

### General characteristics of the study population

**Fig. 1:**
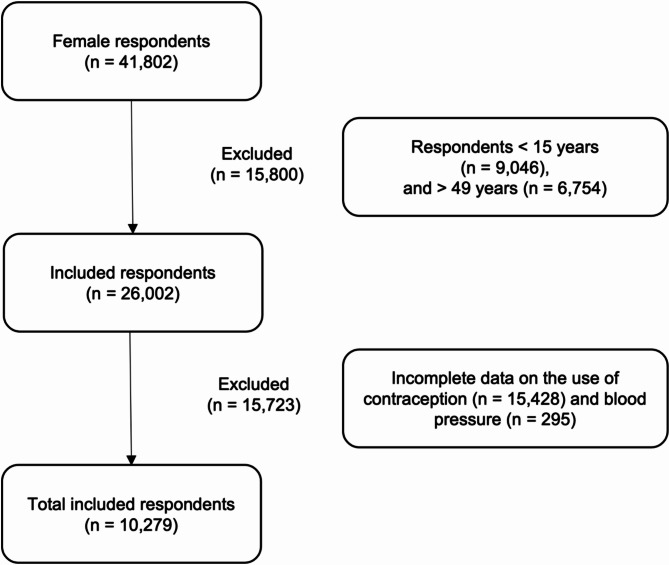
Flow diagram of respondent selection

Overall, out of the 41,802 female respondents registered in IFLS-5, approximately 26,002 met the inclusion criteria. A total of 15,800 respondents were first excluded for being outside the eligible age range of 15–49 years. Subsequently, an additional 15,723 respondents were excluded due to incomplete data on contraceptive use and BP. Therefore, the total number of subjects included in the study was 10,279 respondents. Figure 1 provides the flow chart depicting the details of respondent selection. The majority of participants (40.81%) belonged to the age group of 30–39 years. Of these respondents, 1,150 (11.19%) were OC users, 4,470 (43.49%) were other contraceptive users, and 4,659 (45.33%) were non-users. Additionally, among the respondents included, 1,209 (11.76%) and 1,657 (16.12%) had prehypertension and hypertension, respectively. Table 1 presents the sociodemographic and clinical characteristics of the respondents, while Supplementary Material 2 provides details on the sociodemographic differences among those with missing contraceptive use and BP data.

**Table 1:** Sociodemographic and clinical characteristics of respondents in IFLS-5

Variable	IFLS-5 (2014–2015) (*N* = 10,279)	
	Number (*N*)	Percentages (%)
**Contraceptive use status**		
OC users	1,150	11.2
Other users	4,470	43.5
Non-users	4,659	45.3
**Duration of OC Use**		
0–12 months	237	20.6
12–24 months	51	4.4
> 24 months	362	31.5
Missing data	500	43.5
**Blood pressure categories**		
Normal	7,413	72.1
Prehypertension	1,209	11.8
Hypertension	1,657	16.1
**Age**,** years**		
15–19	288	2.8
20–29	3,127	30.4
30–39	4,195	40.8
40–49	2,669	26.0
**Residential location**		
Rural	5,878	57.2
Urban	4,401	42.8
**Level of education**		
None	226	2.2
Elementary School/Equivalent	2,961	28.8
Junior High School/Equivalent	2,440	23.7
Senior High School/Equivalent	3,273	31.9
University	1,335	13.0
Others	24	0.2
Missing data	20	0.2
**Employment status**		
Not working	4,570	44.5
Working	5,706	55.5
Missing data	3	0.0
**Obesity**		
No	6,586	64.0
Yes	3,665	35.7
Missing data	28	0.3
**History of tobacco use**		
No	10,057	97.8
Yes	217	2.1
Missing data	5	0.1
**Physical activity level**		
Light	3,631	35.3
Moderate	4,262	41.5
Heavy	2,299	22.4
Missing data	87	0.8
**Dietary habits**		
Poor	195	1.9
Borderline	1,328	12.9
Acceptable	8,667	84.3
Missing data	89	0.9
**Depressive symptoms**		
No	7,783	75.7
Yes	2,408	23.4
Missing data	88	0.9
**Comorbidities**		
**Diabetes mellitus**		
No	10,143	98.6
Yes	131	1.3
Missing data	5	0.1
**Cardiovascular diseases**		
No	10,133	98.6
Yes	141	1.3
Missing data	5	0.1
**Dyslipidemia**		
No	9,912	96.4
Yes	362	3.5
Missing data	5	0.1

### Association of oral contraceptive use and prehypertension and hypertension

Among oral contraceptive users, the prevalence of normal BP, prehypertension, and hypertension was 61.39%, 14.43%, and 24.17%, respectively. In comparison, these prevalence rates were 75.15%, 11.45%, and 13.40% among users of other contraceptive methods, and 71.86%, 11.40%, and 16.74% among non-users. The differences across groups were statistically significant (*p* < 0.001).

Statistically significant differences were also observed in age, residential location, levels of education, employment status, obesity, physical activities, dietary habits, diabetes mellitus, CVDs, and dyslipidemia, as shown in Table 2. After adjusting for potential confounders, the use of OCs was significantly associated with increased odds of developing prehypertension and hypertension. The main analysis was conducted using two reference groups: the first comparing OC users to non-users, and the second compared OC users to users of other contraceptive methods. Among OC users versus non-users, the aOR for prehypertension and hypertension were 1.42 (95% CI 1.16–1.73; *p* = 0.001) and 1.72 (95% CI 1.45–2.05; *p* < 0.001), respectively. Similarly, for OC users compared to users of other contraceptive methods, the aOR for prehypertension and hypertension were 1.74 (95% CI: 1.21–2.51; *p* = 0.003) and 1.80 (95% CI: 1.31–2.48; *p* < 0.001), respectively (Table 3). The pseudo-R-squared values of the model were 10.07% and 10.09% for comparisons with non-users and users of other contraceptive methods, respectively.

The results of the sub-analyses were consistent with those of the main analysis, particularly for hypertension. In the first sub-analysis, OC users were compared to non-users, while in the second sub-analysis, OC users were compared to users of other modern contraceptive methods, categorized by the duration of OC use. The odds of hypertension increased with longer durations of OC use in both subgroups, although statistical significance was observed only for durations of 0–12 months and > 24 months. For OC users compared to non-users, the aOR for hypertension were 1.71 (95% CI: 1.18–2.48; *p* = 0.005) for 0–12 months, 1.77 (95% CI: 0.84–3.73; *p* = 0.130) for 12–24 months, and 1.91 (95% CI: 1.45–2.51; *p* < 0.001) for > 24 months (Table 4). Similarly, when compared to users of other modern contraceptive methods, the aOR for hypertension were 1.81 (95% CI: 1.26–2.61; *p* < 0.001) for 0–12 months, 1.97 (95% CI: 0.95–4.09; *p* = 0.068) for 12–24 months, and 2.14 (95% CI: 1.62–2.81; *p* < 0.001) for > 24 months (Table 5). In contrast, the duration of OC use was not associated with prehypertension.

**Table 2:** Bivariate analysis results on IFLS-5

Variable	Blood pressure categories						
	Normal (*N*, %)		Prehypertension (*N*, %)		Hypertension (*N*, %)		*p*-value
Contraceptive use status							
OC user	706	(61.4%)	166	(14.4%)	278	(24.2%)	< 0.001
Other user	3,359	(75.1%)	512	(11.5%)	599	(13.4%)	
Non-user	3,348	(71.9%)	531	(11.4%)	780	(16.7%)	
**Duration of OC Use**							
0–12 month	163	(68.8%)	28	(11.8%)	46	(19.4%)	0.230
12–24 month	35	(68.6%)	5	(9.8%)	11	(21.6%)	
> 24 months	219	(60.5%)	45	(12.4%)	98	(27.1%)	
**Age**,** years**							
15–19	257	(89.2%)	23	(8.0%)	8	(2.8%)	< 0.001*
20–29	2,673	(85.5%)	258	(8.2%)	196	(6.3%)	
30–39	3,120	(74.4%)	479	(11.4%)	596	(14.2%)	
40–49	1,363	(51.1%)	449	(16.8%)	857	(32.1%)	
**Residential location**							
Rural	4,272	(72.7%)	636	(10.8%)	970	(16.5%)	0.002*
Urban	3,141	(71.4%)	573	(13.0%)	687	(15.6%)	
**Level of education**							
None	121	(53.5%)	34	(15.1%)	71	(31.4%)	< 0.001*
Elementary School/Equivalent	1,868	(63.1%)	434	(14.6%)	659	(22.3%)	
Junior High School/Equivalent	1,819	(74.5%)	280	(11.5%)	341	(14.0%)	
Senior High School/Equivalent	2,492	(76.1%)	345	(10.5%)	436	(13.3%)	
University	1,080	(80.9%)	111	(8.3%)	144	(10.8%)	
Others	19	(79.2%)	2	(8.3%)	3	(12.5%)	
**Employment status**							
Not working	3,361	(73.5%)	505	(11.1%)	704	(15.40%)	0.016*
Working	4,051	(71.0%)	702	(12.3%)	953	(16.7%)	
**Obesity**							
No	5,273	(80.1%)	620	(9.4%)	693	(10.5%)	< 0.001*
Yes	2,121	(57.9%)	587	(16.0%)	957	(26.1%)	
**History of tobacco use**							
No	7,253	(72.1%)	1,182	(11.8%)	1,622	(16.1%)	0.951
Yes	158	(72.8%)	24	(11.1%)	35	(16.1%)	
**Physical activity level**							
Light	2,690	(74.1%)	385	(10.6%)	556	(15.3%)	0.008*
Moderate	3,062	(71.9%)	504	(11.8%)	696	(16.3%)	
Heavy	1,605	(69.8%)	306	(13.3%)	388	(16.9)%	
**Dietary habits**							
Poor	129	(66.2%)	25	(12.8%)	41	(21.0%)	0.021*
Borderline	919	(69.2%)	172	(13.0%)	237	(17.8%)	
Acceptable	6,307	(72.8%)	998	(11.5%)	1,362	(15.7%)	
**Depressive symptoms**							
No	5,591	(71.8%)	931	(12.0%)	1,261	(16.2%)	0.308
Yes	1,765	(73.3%)	264	(11.0%)	379	(15.7%)	
**Diabetes mellitus**							
No	7,350	(72.5%)	1,181	(11.6%)	1,612	(15.9%)	< 0.001*
Yes	61	(46.6%)	25	(19.1%)	45	(34.3%)	
**Cardiovascular diseases**							
No	7,326	(72.3%)	1,184	(11.7%)	1,623	(16.0%)	0.006*
Yes	85	(60.3%)	22	(15.6%)	34	(24.1%)	
**Dyslipidemia**							
No	7,220	(72.8%)	1,161	(11.7%)	1,531	(15.5%)	< 0.001*
Yes	191	(52.8%)	45	(12.4%)	126	(34.8%)	

**Table 3:** Binary logistic regression results for prehypertension and hypertension in OC users compared to non-users and other modern contraceptive users

	IFLS-5 (2014–2015)				
	Normal	Prehypertension	*p*-value	Hypertension	*p*-value
***OC users vs. non-users***					
**Non-user**	*Reference*				
**OC users**					
*Crude* OR (95% CI)	*Ref*	1.48 (1.22–1.80)	< 0.001*	1.69 (1.44–1.98)	< 0.001*
*Adjusted* OR^a^ (95% CI)	*Ref*	1.42 (1.16–1.73)	0.001*	1.72 (1.45–2.05)	< 0.001*
***OC users vs. other users***					
**Other users**	*Reference*				
**OC users**					
*Crude* OR (95% CI)	*Ref*	1.54 (1.27–1.87)	< 0.001*	2.21 (1.87–2.60)	< 0.001*
*Adjusted* OR^a^(95% CI)	*Ref*	1.74 (1.21–2.51)	0.003*	1.80 (1.31–2.48)	< 0.001*

^a^adjusted to age, residential location, level of education, employment status, obesity, physical activity levels, dietary habits, diabetes mellitus, CVDs, and dyslipidemia

∗*p* < 0.05 at the 5% level of significance

**Table 4:** Sub-analysis for prehypertension and hypertension by duration of OC use, compared to non-users

	IFLS-5 (2014–2015)				
	Normal	Prehypertension	*p*-value	Hypertension	*p*-value
**Non-user**	*Reference*				
**0–12 month**					
*Crude* OR (95% CI)	*Ref*	1.08 (0.72–1.63)	0.704	1.21 (0.87–1.70)	0.264
*Adjusted* OR^a^ (95% CI)	*Ref*	1.30 (0.847–1.99)	0.232	1.71 (1.18–2.48)	0.005*
**12–24 month**					
*Crude* OR (95% CI)	*Ref*	0.90 (0.35–2.31)	0.828	1.35 (0.68–2.67)	0.390
*Adjusted* OR ^a^(95% CI)	*Ref*	0.98 (0.37–2.57)	0.967	1.77 (0.84–3.73)	0.130
**> 24 month**					
*Crude* OR (95% CI)	*Ref*	1.30 (0.93–1.81)	0.128	1.92 (1.49–2.47)	< 0.001*
*Adjusted* OR^a^ (95% CI)	*Ref*	1.24 (0.87–1.75)	0.232	1.91 (1.45–2.51)	< 0.001*

OC: oral contraceptive

^a^adjusted to age, residential location, level of education, employment status, obesity, physical activity levels, dietary habits, diabetes mellitus, CVDs, and dyslipidemia

∗*p* < 0.05 at the 5% level of significance

**Table 5:** Sub-analysis for prehypertension and hypertension by duration of OC use, compared to other contraceptive users

	IFLS-5 (2014–2015)				
	Normal	Prehypertension	*p*-value	Hypertension	*p*-value
**Non-user**	*Reference*				
**0–12 month**					
*Crude* OR (95% CI)	*Ref*	1.13 (0.75–1.70)	0.569	1.58 (1.12–2.22)	0.008*
*Adjusted* OR^a^ (95% CI)	*Ref*	1.18 (0.77–1.80)	0.440	1.81 (1.26–2.61)	0.001*
**12–24 month**					
*Crude* OR (95% CI)	*Ref*	0.94 (0.37–2.40)	0.893	1.76 (0.89–3.49)	0.104
*Adjusted* OR ^a^(95% CI)	*Ref*	0.91 (0.35–2.36)	0.844	1.97 (0.95–4.09)	0.068
**> 24 month**					
*Crude* OR (95% CI)	*Ref*	1.35 (0.97–1.88)	0.080	2.51 (1.95–3.2)	< 0.001*
*Adjusted* OR^a^ (95% CI)	*Ref*	1.22 (0.86–1.72)	0.260	2.14 (1.62–2.81)	< 0.001*

OC: oral contraceptive

^a^adjusted to age, residential location, level of education, employment status, obesity, physical activity levels, dietary habits, diabetes mellitus, CVDs, and dyslipidemia

∗*p* < 0.05 at the 5% level of significance

## Discussion

The findings of this study indicated that the use of OCs may associated with an increased risk of developing prehypertension and hypertension in Indonesia, with the risk of hypertension increasing with longer durations of OC use. After adjusting for potential confounders, OC users exhibited higher odds of developing prehypertension and hypertension compared to both non-users and users of other modern contraceptive methods.

Evidence of the association between OC use and the increased risk of elevated BP has been extensively documented in various population. Several large population studies from the United States (US), United Kingdom (UK), and Germany in the 1990s [38–40], and, more recently from Korea, have all reported significant but moderate elevations in BP among OC users [14]. The Nurses’ Health Study, for instance, found that current OC users had slightly elevated SBP levels (0.7 mmHg)and DBP (0.4 mmHg) compared to non-users, after adjusting for various confounding factors [38]. Nationally representative studies conducted in the UK, Germany, and Korea observed even more significant increases in BP among OC users, with SBP rising by 2.6 to 5.8 mmHg and DBP rising by 1.8 to 3.6 mmHg [14, 39, 40]. These consistent findings across different populations reinforce the robustness of the link between OC use and BP elevation. Similarly, a recent retrospective cohort study in Brazil reported a higher prevalence of high BP, dyslipidemia, and insulin resistance among adolescent OC users (ages 14–17) compared to non-users [41]. However, some discrepancies exist in the literature. A previous Australian study found no significant association between past hormonal contraceptive use and high BP in postmenopausal women [42], suggesting that the effects of OC use on BP may vary by age group and hormonal exposure duration.

The mechanisms underlying BP elevation due to OC use are complex and likely involve multiple interconnected pathways. The estrogen and progestin components of OCs likely interfere with the renin-angiotensin-aldosterone system, a key regulator of BP [43, 44]. Estrogen stimulates the production of angiotensinogen, renin, angiotensin II, and aldosterone, leading to chronic salt retention, increased blood volume, and subsequent BP elevation. Furthermore, angiotensin II promotes oxidative stress by activating NADPH oxidase, which reduces nitric oxide availability, impairs endothelial function, and may contribute to hypertension [43].

Additionally, pre-existing CVD risk factors may exacerbate the BP elevations caused by OC use. Individuals with a family history of hypertension, advanced age, or obesity are found to have significantly higher risks of developing hypertension compared to those without these risk factors [45–47]. Our study also found statistically significant differences in CVD comorbidities between normotensive and hypertensive groups, indicating that underlying CVD risk factors may compound the hypertensive effects of OCs. Although the design of the study limits the ability to definitively attribute this association to OCs alone, the observed differences suggest that CVDs may compound the hypertensive effects of OC use, particularly in women with underlying risk factors.

The duration of OC use appears to be a critical factor influencing hypertension risk. Findings from the Korea National Health and Nutrition Examination Surveys (KNHANES) demonstrated a cumulative risk of hypertension with each additional year of OC use, with the highest risk observed in women who used OCs for two or more years. Our study supports these findings, reinforcing that prolonged OC use is a significant risk factor for hypertension. Based on a previous study, OC use for more than 24 months has been associated with more than twofold increased likelihood of developing prehypertension [17]. This has important clinical implications, suggesting that the duration of hormonal contraceptive use should be closely monitored in women at risk for hypertension.

Notably, different formulations of OCs may have varying effects on BP. Combined OCs, which contain both estrogen and progestin, have been associated with increased hypertension risk due to their earlier introduction and greater efficacy compared to progestin-only pills [14, 15, 45, 48, 49]. Although more recent generations of OCs have been developed with lower doses of estrogen and modified progestin components to minimize cardiovascular side effects, concerns about their impact on BP persist. While studies have demonstrated that newer generations of OCs have a reduced impact on BP compared to earlier versions, conflicting evidence continues to emerge about their influence on other cardiovascular risk factors, including metabolic and hemodynamic parameters [14, 50]. Our findings contribute to the growing body of evidence linking OC use to increased BP and highlight the importance of monitoring BP in OC users, particularly those with prolonged exposure or pre-existing cardiovascular risk factors.

Moreover, studies examining gender-specific risk factors for hypertension have highlighted the higher prevalence of hypertension among women compared to men in Indonesia, as shown in data from the Indonesian Family Life Survey 4 (IFLS-4) [51]. This emphasizes the importance of investigating female-specific risk factors, such as OC use, that may contribute to this disparity. While hormonal changes associated with both OC use and pregnancy can influence vascular and hemodynamic responses, the underlying mechanisms are complex and multifactorial, involving factors such as immune responses and weight gain during pregnancy. Furthermore, previous studies suggest that the hypertension risk associated with OC use may not be permanent, as research indicates that blood pressure levels could return to baseline after discontinuation [38]. Given these considerations, it is essential to balance concerns about OC-related hypertension with the broader implications of contraceptive access, as restricting options could lead to an increased risk of unintended pregnancies, particularly in settings where reproductive choices are limited.

Inevitably, this study has several limitations. First, its cross-sectional design restricts the ability to establish causality between OC use and changes in BP. Therefore, it is crucial to emphasize that cross-sectional studies provide only a snapshot in time and do not capture how these variables interact over an extended period. This lack of temporal data hinders our ability to draw definitive conclusions regarding the directionality of the relationship between OC use and changes in BP. Future longitudinal studies are necessary to investigate the effects of OC use on BP over time. Second, this study did not account for important confounding variables, such as the specific type and dosage of OCs and the family history of hypertension, as these variables were unavailable in the IFLS-5 dataset, limiting a more detailed analysis. These constraints are common in large-scale surveys, but future studies with more comprehensive data could provide deeper insights into the effects of different contraceptive methods on BP. Previous studies have shown that genetic predisposition plays a significant role in BP regulation. Individuals with a family history of hypertension are at a higher risk of developing elevated BP due to inherited genetic factors and shared environmental [52]. By not accounting for this variable, the study may not fully capture the independent effect of OC use on BP. This omission could lead to an over or underestimation of the association between OC use and elevated BP, as some of the observed effects may be partially attributable to genetic predisposition rather than the contraceptive itself. Moreover, the dataset does not provide information to differentiate between combined OCs and progestin-only pills. However, based on the timing of the survey administration (prior to 2014), combined OCs were the predominant type of oral contraceptives in use, while progestin-only pills, commonly referred to as “mini-pills,” were less commonly used [53]. While our study does not differentiate between combined OC and progestin-only pills due to data limitations, this distinction is important for understanding the varying cardiovascular risks associated with different contraceptive formulations. A higher proportion of women with secondary and tertiary education were also observed in this study compared to national estimates from Statistics Indonesia (BPS) in 2014, where 15.78% of women aged 15 years and over had completed high school and 6.89% had attained university-level education [54]. This suggests that our respondents may be more educated than the general Indonesian population, potentially influencing health awareness, contraceptive choices, and access to healthcare services. As education level can impact health-related decision-making, this difference should be considered when interpreting our findings. In addition, we recognize that societal changes over the past decade, along with potential shifts in behavior and economic factors, may affect the relevance of the IFLS-5 dataset in the context of current demographic and health conditions. However, it remains an important resource due to its comprehensive national coverage and the foundational insights it provides into demographic and health trends. We suggested that future research utilize more recent data for comparison.

Some variables, including tobacco use history, physical activity levels, dietary habits, and OC use, were self-reported, introducing the potential for response bias or inaccuracies. The lack of biochemical validation for these variables further limits the ability to ensure their accuracy. Specifically, for OC use, while the question clearly defined its purpose for pregnancy prevention, it is also used for other medical conditions, such as polycystic ovarian syndrome (PCOS). As a result, there is a potential misunderstanding, where respondents using contraception for non-pregnancy-related reasons may have answered ‘yes,’ leading to an overestimation of OC use. Furthermore, the high proportion of missing data on contraceptive use likely reflects cultural and social sensitivities in Indonesia, where discussing reproductive health remains somewhat taboo in certain communities [55]. As shown in Supplementary Material 1, there are significant differences between participants included in the analysis and those excluded due to missing contraceptive use data. This highlights the potential for selection bias, and therefore, our findings should be interpreted with caution. Similar trends have been observed in other surveys on reproductive health, where self-reported contraceptive use data often suffer from underreporting due to privacy concerns, recall bias, or social desirability bias [56].

Another limitation is the broad categorization of various modern contraceptive users as a single reference group, combining hormonal (e.g., injections, implants) and non-hormonal methods (e.g., condoms). Since hormonal methods may have varying effects on BP, this grouping limits the ability to isolate the specific impact of OC use. Future research should consider stratifying contraceptive methods into more specific categories for a clearer comparison. Lastly, the relatively small pseudo-R-squared value observed in this study indicates that additional variables may need to be considered to better explain the relationship between OC use and the increased risk of elevated BP. Future studies are needed to estimate the population-attributable fractions and to conduct further subgroup analyses aimed at identifying which women are at the highest risk of hypertension.

Despite these limitations, the study has notable strengths. By utilizing data from IFLS, the analysis was based on a large, nationally representative sample of Indonesian women of childbearing age. This allows for the generalizability of the findings and broader implications across the Indonesian population. Moreover, this study uniquely examines not only the association between OC use and the incidence of hypertension but also extends the analysis to prehypertension. This offers a more comprehensive view of how OC use may influence BP across a spectrum of risk, from early-stage prehypertension to hypertension. Furthermore, by examining the duration of OC use, the study provides a deeper understanding of its potential role in the increased risk of elevated BP.

Given that OCs are the most commonly used contraceptive method in Indonesia, these findings hold significant public health and clinical implications. The results underscore the importance of healthcare providers informing patients of the potential link between OC use and an increased risk of both prehypertension and hypertension. This information is particularly vital for women who are more susceptible, such as those with a family history of hypertension or other cardiovascular risk factors. Ensuring that these women are aware of the risks enables them to make more informed decisions about their contraceptive options. Additionally, for women already diagnosed with hypertension or those at high risk, healthcare providers should consider discussing alternative contraceptive methods that carry a lower risk of raising BP. This could include non-hormonal options or progestin-only pills, which have been shown to have a lesser impact on cardiovascular health. Educating patients about the potential risks of OC use while also providing preventive strategies such as regular BP monitoring and lifestyle modifications, can help mitigate adverse health outcomes and promote better overall cardiovascular management.

## Conclusion

This study demonstrates a statistically significant association between OC use and an increased risk of both prehypertension and hypertension among Indonesian women of reproductive age. Analysis of IFLS-5 data revealed that OC users were over 1.5 times more likely to develop these conditions compared to non-users and users of other modern contraceptive methods. Furthermore, the risk of hypertension was found to increase with longer durations of OC use. These findings highlight the potential cardiovascular risks associated with OC use, emphasizing the need for healthcare providers to carefully consider these risks when prescribing contraceptives, particularly for women with pre-existing cardiovascular risk factors.

## Electronic supplementary material

Below is the link to the electronic supplementary material.

Supplementary Material 1

Supplementary Material 2

## Data Availability

Data is provided within the manuscript or supplementary materials. All data generated or analyzed during this study are included in this published article and availabe online at https://www.rand.org/well-being/social-and-behavioral-policy/data/FLS/IFLS.html.

## Abbreviations

OCs: Oral contraceptives
IFLS-5: Indonesian Family Life Survey 5
OR: Odds ratios
CI: Confidence intervals
CVDs: Cardiovascular diseases
SBP: Systolic blood pressure
DBP: Diastolic blood pressure
IUD: Intrauterine devices
BMI: Body Mass Index
IPAQ-SF: International Physical Activity Questionnaire-Short Form (IPAQ-SF)
