# Supplementary material for: The use of oral contraceptives and the risks of developing prehypertension and hypertension in women of reproductive age: findings from a population-based survey in Indonesia
Source: BMC Public Health. 2025 Apr 24;25:1524. doi: 10.1186/s12889-025-22686-4 (PMC12020133; doi:10.1186/s12889-025-22686-4)
Supplement: Supplementary file 1 — Supplementary Material 1 [file 12889_2025_22686_MOESM1_ESM.doc]

Title: The Use of Oral Contraceptives and the Risks of Developing Prehypertension and Hypertension in Women of Reproductive Age: Findings from Multicenter Population-Based Survey in Indonesia

STROBE Statement—Checklist of items that should be included in reports of ***cross-sectional studies***

|  | Item No | Recommendation | Checklist | Note |
| --- | --- | --- | --- | --- |
| **Title and abstract** | 1 | (*a*) Indicate the study’s design with a commonly used term in the title or the abstract | Yes | *See title* |
|  |  | (*b*) Provide in the abstract an informative and balanced summary of what was done and what was found | Yes | *See abstract* |
| Introduction | | |  |  |
| Background/rationale | 2 | Explain the scientific background and rationale for the investigation being reported | Yes | *See introduction paragraph 2 and 3* |
| Objectives | 3 | State specific objectives, including any prespecified hypotheses | Yes | *See introduction paragraph 3* |
| Methods | | |  |  |
| Study design | 4 | Present key elements of study design early in the paper | Yes | *See ‘Data Sources and Study Design’, paragraph 1.* |
| Setting | 5 | Describe the setting, locations, and relevant dates, including periods of recruitment, exposure, follow-up, and data collection | Yes | *See ‘Data Sources and Study Design’, paragraph 1.* |
| Participants | 6 | (*a*) Give the eligibility criteria, and the sources and methods of selection of participants |  | The study included women of reproductive age (15-49 years) with complete data on oral contraceptives, blood pressure, and sociodemographic characteristics. Exclusions were made for those with incomplete data on blood pressure measurements, contraceptive use, and pregnancy.  *(See ‘Data Sources and Study Design’, paragraph 2)* |
| Variables | 7 | Clearly define all outcomes, exposures, predictors, potential confounders, and effect modifiers. Give diagnostic criteria, if applicable | Yes | *See ‘Variable classification’, paragraph 1.* |
| Data sources/ measurement | 8* | For each variable of interest, give sources of data and details of methods of assessment (measurement). Describe comparability of assessment methods if there is more than one group | Yes | **Source of Data:**  Data was obtained from the Indonesian Family Life Survey (IFLS) 5 which conducted in 2014 to 2015. *(See ‘Data Sources and Study Design’, paragraph 1).*  **Details of measurement method:**  BP data collected through direct measurements or medical diagnosis; OC usage via questionnaire; sociodemographics from questionnaires, direct measurement, IPAQ scoring. *(See Variable Classification section).*  **Comparability between groups:**  Respondents grouped by OC use (users, non-users) and by BP status (prehypertension, hypertension). *(See Variable Classification section).* |
| Bias | 9 | Describe any efforts to address potential sources of bias | Yes | The inclusion of sociodemographic and comorbidities as potential confounding factors (see *See Variable Classification section).* |
| Study size | 10 | Explain how the study size was arrived at | Yes | The exact sample size is illustrated in Figure 1 (Flow diagram of respondent selection). This figure provides details on the selection process of respondents, which can be further explored in the *Results section under "general characteristics of the study population”.* |
| Quantitative variables | 11 | Explain how quantitative variables were handled in the analyses. If applicable, describe which groupings were chosen and why | Yes | Blood pressure (BP) is the dependent variable, while the use of oral contraceptives (OCs) is the independent variable. The sociodemographic factors considered as covariates or control variables. These sociodemographic factors help to adjust for potential confounding in analysis.  *(See Variable Classification section).* |
| Statistical methods | 12 | (*a*) Describe all statistical methods, including those used to control for confounding | Yes | Descriptive analyses were conducted to determine the distribution and characteristics of respondents’ data on each variable, reported as total numbers (N) and percentages (%). Sociodemographic characteristics and comorbidities were compared between three BP level groups (normal, prehypertension, and hypertension) with a chi-square test. Variables with a significance level of *p* < 0.25 in univariate analyses were included in the multivariate analysis. Binary logistic regression was performed to obtain the crude and adjusted odds ratio (cOR and aOR, respectively), 95% CI (confidence interval), and p-value. R-squared was also estimated to indicate the extent to which the combination of independent variables simultaneously explains the variation in the dependent variable  *(See statistical analysis section).* |
|  |  | (*b*) Describe any methods used to examine subgroups and interactions | Yes | a sub-analysis was conducted to evaluate the association between prehypertension and hypertension with the duration of OC use. This analysis was performed within a single binary logistic regression model, where OC duration was treated as a categorical variable (short-term, medium-term, long-term). Comparisons were made between OC users, users of other modern contraceptive methods, and non-users.  *(See statistical analysis section).* |
|  |  | (*c*) Explain how missing data were addressed | Yes | *See Supplementary material 2 and Table 1* |
|  |  | (*d*) If applicable, describe analytical methods taking account of sampling strategy | Not applicable | - |
|  |  | (*e*) Describe any sensitivity analyses | Not applicable | - |
| Results | | |  |  |
| Participants | 13* | (a) Report numbers of individuals at each stage of study—eg numbers potentially eligible, examined for eligibility, confirmed eligible, included in the study, completing follow-up, and analysed | Yes | - *Potentially eligible: 41,802* - *Examined for eligibility: 15,723* - *Confirmed eligible: 10,279* - *Included in the study: 10,279*   *(See Results ‘General characteristics of the study population’)* |
|  |  | (b) Give reasons for non-participation at each stage | Yes | First stage: Respondent above 49 years old and incomplete data on the use of OCs and BP.  Second stage: Respondents were excluded due to missing data on the use of contraception and BP.  *(See Results ‘General characteristics of the study population’)* |
|  |  | (c) Consider use of a flow diagram | Yes | A flow diagram illustrating participant selection and reasons for non-participation is included in Figure 1.  *(See Results ‘General characteristics of the study population’)* |
| Descriptive data | 14* | (a) Give characteristics of study participants (eg demographic, clinical, social) and information on exposures and potential confounders | Yes | The sociodemographic characteristics and clinical conditions of the respondents are shown in Table 1.  *(See Results ‘General characteristics of the study population’)* |
|  |  | (b) Indicate number of participants with missing data for each variable of interest | Yes | A flow diagram illustrating number of participants with missing data for each variable of interest (Figure 1).  *(See Results ‘General characteristics of the study population’)* |
| Outcome data | 15* | Report numbers of outcome events or summary measures | Yes | *(See Results ‘Association of Oral Contraceptive Use and Prehypertension and Hypertension’)* |
| Main results | 16 | (*a*) Give unadjusted estimates and, if applicable, confounder-adjusted estimates and their precision (eg, 95% confidence interval). Make clear which confounders were adjusted for and why they were included | Yes | The main analysis was conducted using two reference groups: the first comparing OC users to non-users, and the second compared OC users to users of other contraceptive methods. Among OC users versus non-users, the aOR for prehypertension and hypertension were 1.42 (95% CI 1.16–1.73; *p* = 0.001) and 1.72 (95% CI 1.45–2.05; *p* < 0.001), respectively. Similarly, for OC users compared to users of other contraceptive methods, the aOR for prehypertension and hypertension were 1.74 (95% CI: 1.21–2.51; *p* = 0.003) and 1.80 (95% CI: 1.31–2.48; *p* < 0.001), respectively. This result is summarized in Table 3.  *(See Results ‘Association of Oral Contraceptive Use and Prehypertension and Hypertension’, paragraph 2).* |
|  |  | (*b*) Report category boundaries when continuous variables were categorized | Not applicable |  |
|  |  | (*c*) If relevant, consider translating estimates of relative risk into absolute risk for a meaningful time period | Not applicable | No absolute risk estimates were given. |
| Other analyses | 17 | Report other analyses done—eg analyses of subgroups and interactions, and sensitivity analyses | Yes | The results of the sub-analyses were consistent with those of the main analysis, particularly for hypertension. In the first sub-analysis, OC users were compared to non-users, while in the second sub-analysis, OC users were compared to users of other modern contraceptive methods, categorized by the duration of OC use. The odds of hypertension increased with longer durations of OC use in both subgroups, although statistical significance was observed only for durations of 0–12 months and > 24 months. For OC users compared to non-users, the aOR for hypertension were 1.71 (95% CI: 1.18–2.48; *p* = 0.005) for 0–12 months, 1.77 (95% CI: 0.84–3.73; *p* = 0.130) for 12–24 months, and 1.91 (95% CI: 1.45–2.51; *p* < 0.001) for > 24 months (Table 4). Similarly, when compared to users of other modern contraceptive methods, the aOR for hypertension were 1.81 (95% CI: 1.26–2.61; *p* < 0.001) for 0–12 months, 1.97 (95% CI: 0.95–4.09; *p* = 0.068) for 12–24 months, and 2.14 (95% CI: 1.62–2.81; *p* < 0.001) for > 24 months (Table 5) *(See Results ‘Association of Oral Contraceptive Use and Prehypertension and Hypertension’, paragraph 3).* |
| Discussion | | |  |  |
| Key results | 18 | Summarise key results with reference to study objectives | Yes | *See Discussion, paragraph 1.* |
| Limitations | 19 | Discuss limitations of the study, taking into account sources of potential bias or imprecision. Discuss both direction and magnitude of any potential bias | Yes | *See Discussion, paragraph 6.* |
| Interpretation | 20 | Give a cautious overall interpretation of results considering objectives, limitations, multiplicity of analyses, results from similar studies, and other relevant evidence | Yes | **Overall interpretation:**  *See Discussion, paragraph 1.*  **Limitations:**  *See Discussion, paragraph 6.*  **Results from similar studies:**  *See Discussion, paragraph 2.*  **Other relevant evidence:**  *See Discussion, paragraph 4, 5.* |
| Generalisability | 21 | Discuss the generalisability (external validity) of the study results | Yes | *See Discussion* |
| Other information | | |  |  |
| Funding | 22 | Give the source of funding and the role of the funders for the present study and, if applicable, for the original study on which the present article is based | Yes | *See funding* |

*Give information separately for exposed and unexposed groups.

**Note:** An Explanation and Elaboration article discusses each checklist item and gives methodological background and published examples of transparent reporting. The STROBE checklist is best used in conjunction with this article (freely available on the Web sites of PLoS Medicine at http://www.plosmedicine.org/, Annals of Internal Medicine at http://www.annals.org/, and Epidemiology at http://www.epidem.com/). Information on the STROBE Initiative is available at www.strobe-statement.org.
