# Supplementary material for: The use of oral contraceptives and the risks of developing prehypertension and hypertension in women of reproductive age: findings from a population-based survey in Indonesia
Source: BMC Public Health. 2025 Apr 24;25:1524. doi: 10.1186/s12889-025-22686-4 (PMC12020133; doi:10.1186/s12889-025-22686-4)
Supplement: Supplementary file 2 — Supplementary Material 2 [file 12889_2025_22686_MOESM2_ESM.docx]

Table 1. Bivariate analysis of sociodemographic differences between included and excluded participants in the study based on data from IFLS-5.

| **Variable** | **Data categories** | | | | |
| --- | --- | --- | --- | --- | --- |
|  | **Included**  **(N, %)** | | **Excluded***  **(N, %)** | | **p-value** |
| **Age, years** |  | |  | |  |
| 15-19 | 288 | 2.8 | 2,943 | 19.08 | <0.001 |
| 20-29 | 3,127 | 30.42 | 4,893 | 31.72 |  |
| 30-39 | 4,195 | 40.81 | 5,206 | 33.74 |  |
| 40-49 | 2,669 | 25.97 | 2,386 | 15.47 |  |
| **Residential location** |  |  |  |  |  |
| Rural | 5,878 | 57.18 | 9,443 | 61.21 | <0.001 |
| Urban | 4,401 | 42.82 | 5,985 | 38.79 |  |
| **Level of education** |  |  |  |  |  |
| None | 226 | 2.20 | 223 | 1.45 | <0.001 |
| Elementary School/Equivalent | 2,961 | 28.81 | 3,010 | 19.51 |  |
| Junior High School/Equivalent | 2,440 | 23.74 | 2,840 | 18.41 |  |
| Senior High School/Equivalent | 3,273 | 31.84 | 5,637 | 36.54 |  |
| University | 1,335 | 12.99 | 3,011 | 19.52 |  |
| Others | 24 | 0.23 | 55 | 0.36 |  |
| Missing data | 20 | 0.19 | 652 | 4.23 |  |
| **Employment status** |  |  |  |  |  |
| Not working | 4,570 | 44.46 | 1,850 | 11.99 | <0.001 |
| Working | 5,706 | 55.51 | 1,243 | 8.06 |  |
| Missing data | 3 | 0.03 | 12,335 | 79.95 |  |
| **Obesity** |  |  |  |  |  |
| No | 6,586 | 64.07 | 2,517 | 16.31 | <0.001 |
| Yes | 3,665 | 35.66 | 379 | 2.46 |  |
| Missing data | 28 | 0.27 | 12,532 | 81.23 |  |
| **Diabetes mellitus** |  |  |  |  |  |
| No | 10,143 | 98.68 | 3,044 | 19.73 | <0.001 |
| Yes | 131 | 1.27 | 7 | 0.05 |  |
| Missing data | 5 | 0.05 | 12,377 | 80.22 |  |
| **Physical activity level** |  |  |  |  |  |
| Light | 3,631 | 35.32 | 1,239 | 8.03 | <0.001 |
| Moderate | 4,262 | 41.46 | 1,119 | 7.25 |  |
| Heavy | 2,299 | 22.37 | 434 | 2.81 |  |
| Missing data | 87 | 0.85 | 12,636 | 81.90 |  |
| **Dietary habits** |  |  |  |  |  |
| Poor | 195 | 1.90 | 36 | 0.23 | <0.001 |
| Borderline | 1,328 | 12.92 | 301 | 1.95 |  |
| Acceptable | 8,667 | 84.32 | 2,439 | 15.81 |  |
| Missing data | 89 | 0.87 | 12,652 | 82.01 |  |
| **Cardiovascular diseases** | | | | | |
| No | 10,133 | 98.58 | 3,023 | 19.59 | <0.001 |
| Yes | 141 | 1.37 | 27 | 0.18 |  |
| Missing data | 5 | 0.05 | 1 | 0.01 |  |
| **Dyslipidemia** |  |  |  |  |  |
| No | 9,912 | 96.43 | 3,015 | 19.54 | <0.001 |
| Yes | 362 | 3.52 | 35 | 0.23 |  |
| Missing data | 5 | 0.05 | 12,378 | 80.23 |  |
| **History of tobacco use** | | | | | |
| No | 10,057 | 97.84 | 3,021 | 19.58 | <0.001 |
| Yes | 217 | 2.11 | 35 | 0.23 |  |
| Missing data | 5 | 0.05 | 12,372 | 80.19 |  |
| **Depressive symptoms** | | | | | |
| No | 7,783 | 75.72 | 1,886 | 12.22 | <0.001 |
| Yes | 2,408 | 23.43 | 900 | 5.83 |  |
| Missing data | 88 | 0.86 | 12,642 | 81.94 |  |
| **Blood Pressure** | | | | | |
| Normal | 7,413 | 72.12 | 2,534 | 16.42 | <0.001 |
| Prehypertension | 1,209 | 11.76 | 198 | 1.28 |  |
| Hypertension | 1,657 | 16.12 | 154 | 1.00 |  |
| Missing data | 0 | 0 | 12,542 | 81.29 |  |

*No data on OC use and BP

IFLS-5: Indonesia Family Life Survey-5; OC: Oral contraceptive; BP: Blood pressure
